# *Streptococcus pneumoniae* Translocates into the Myocardium and Forms Unique Microlesions That Disrupt Cardiac Function

**DOI:** 10.1371/journal.ppat.1004383

**Published:** 2014-09-18

**Authors:** Armand O. Brown, Beth Mann, Geli Gao, Jane S. Hankins, Jessica Humann, Jonathan Giardina, Paola Faverio, Marcos I. Restrepo, Ganesh V. Halade, Eric M. Mortensen, Merry L. Lindsey, Martha Hanes, Kyle I. Happel, Steve Nelson, Gregory J. Bagby, Jose A. Lorent, Pablo Cardinal, Rosario Granados, Andres Esteban, Claude J. LeSaux, Elaine I. Tuomanen, Carlos J. Orihuela

**Affiliations:** 1 Dept. of Microbiology and Immunology, University of Texas Health Science Center at San Antonio, San Antonio, Texas, United States of America; 2 Dept. of Infectious Diseases, St. Jude Children's Research Hospital, Memphis, Tennessee, United States of America; 3 Dept. of Hematology, St. Jude Children's Research Hospital, Memphis, Tennessee, United States of America; 4 University of Milan Bicocca and Dept. of Respiratory Medicine, San Gerardo Hospital, Monza, Italy; 5 Dept. of Medicine, South Texas Veterans Health Care System and University of Texas Health Science Center at San Antonio, San Antonio, Texas, United States of America; 6 Division of Cardiovascular Disease, Dept. of Medicine, The University of Alabama at Birmingham, Birmingham, Alabama, United States of America; 7 Medical Service, Veterans Affairs North Texas Health Care System and Dept. of Internal Medicine and Clinical Sciences, University of Texas Southwestern Medical Center, Dallas, Texas, United States of America; 8 Dept. of Physiology and Biophysics University of Mississippi Medical Center, Jackson, Mississippi, United States of America; 9 Dept. of Laboratory Animal Resources. University of Texas Health Science Center at San Antonio, San Antonio, Texas, United States of America; 10 Dept. of Physiology and Section of Pulmonary/Critical Care Medicine. Louisiana State University Health Sciences Center, New Orleans, Louisiana, United States of America; 11 CIBER de Enfermedades Respiratorias, Hospital Universitario de Getafe, Madrid, Spain; 12 Division of Cardiology, Dept. of Medicine, University of Texas Health Science Center at San Antonio, San Antonio, Texas, United States of America; Boston Children's Hospital, United States of America

## Abstract

Hospitalization of the elderly for invasive pneumococcal disease is frequently accompanied by the occurrence of an adverse cardiac event; these are primarily new or worsened heart failure and cardiac arrhythmia. Herein, we describe previously unrecognized microscopic lesions (microlesions) formed within the myocardium of mice, rhesus macaques, and humans during bacteremic *Streptococcus pneumoniae* infection. In mice, invasive pneumococcal disease (IPD) severity correlated with levels of serum troponin, a marker for cardiac damage, the development of aberrant cardiac electrophysiology, and the number and size of cardiac microlesions. Microlesions were prominent in the ventricles, vacuolar in appearance with extracellular pneumococci, and remarkable due to the absence of infiltrating immune cells. The pore-forming toxin pneumolysin was required for microlesion formation but Interleukin-1β was not detected at the microlesion site ruling out pneumolysin-mediated pyroptosis as a cause of cell death. Antibiotic treatment resulted in maturing of the lesions over one week with robust immune cell infiltration and collagen deposition suggestive of long-term cardiac scarring. Bacterial translocation into the heart tissue required the pneumococcal adhesin CbpA and the host ligands Laminin receptor (LR) and Platelet-activating factor receptor. Immunization of mice with a fusion construct of CbpA or the LR binding domain of CbpA with the pneumolysin toxoid L460D protected against microlesion formation. We conclude that microlesion formation may contribute to the acute and long-term adverse cardiac events seen in humans with IPD.

## Author Summary

Hospitalization for community-acquired pneumonia carries a documented risk for adverse cardiac events. These occur during infection and contribute to elevated mortality rates in convalescent individuals up to 1 year thereafter. We describe a previously unrecognized pathogenic mechanism by which *Streptococcus pneumoniae*, the leading cause of community-acquired pneumonia, causes direct cardiotoxicity and forms microscopic bacteria-filled lesions within the heart. Microlesions were detected in experimentally infected mice and rhesus macaques, as well as in heart sections from humans who succumbed to invasive pneumococcal disease (IPD). Cardiac microlesion formation required interaction of the bacterial adhesin CbpA with host Laminin receptor and bacterial cell wall with Platelet-activating factor receptor. Microlesion formation also required the pore-forming toxin pneumolysin. When infected mice were rescued with antibiotics, we observed robust signs of collagen deposition at former lesion sites. Thus, microlesions and the scarring that occurs thereafter may explain why adverse cardiac events occur during and following IPD.

## Introduction

Severe community-acquired pneumonia (CAP) carries an extensively documented risk for adverse cardiac events such as congestive heart failure, arrhythmias, and myocardial infarction. A meta-analysis of 19 observational studies determined that the pooled incidence rate for cardiac complications during hospitalization for CAP is approximately 18% [1]. Risk for cardiac complications is greatest immediately following the diagnosis of pneumonia; with approximately 90% of cardiac events occurring within the first 7 days and >50% occurring within the first 24 h [2], [3]. In one study by Corrales-Medina *et al.* of cardiac complications during pneumonia, congestive heart failure occurred in 21%, arrhythmias occurred in 10%, and myocardial infarction occurred in 3% of hospitalized adults. In contrast, these distinct complications occurred in only 1.4%, 1.0% and 0.1% of outpatients, respectively, indicating that disease severity at time of hospital presentation is a significant risk factor. Cardiac complications were implicated as a direct or underlying cause of death in 27% of the pneumonia-associated deaths. Furthermore, death within 30 days of pneumonia onset was up to five times greater in patients who experienced an adverse cardiac event than among those who did not [2]. Importantly, elevated mortality risk in individuals with CAP persists long-after disease resolution. Kaplan *et al.* demonstrated that the 1-year mortality rate in CAP-convalescent individuals to be 2.69-fold higher than that of the general population and 1.93-fold higher than individuals hospitalized for all other reasons [4]. *Streptococcus pneumoniae* (the pneumococcus), is the most common cause of CAP and sepsis [5], and has been directly associated with an adverse cardiac event in 19.4% of 170 admitted adult patients [6]. Thus, adverse cardiac events contribute in a significant fashion to the overall morbidity and mortality that is associated with adult bacterial pneumonia. This includes during pneumococcal infection, the most prevalent setting for CAP.

Acute bacterial pneumonia stresses the heart by increasing myocardial oxygen demand at a time when oxygenation is compromised by ventilation-perfusion mismatch. Pneumonia and the resulting invasive bacterial disease also raise circulating levels of pro-inflammatory cytokines, which promote thrombogenesis and suppress ventricular function [7]. Notably, engagement of Toll-like receptors (TLR)-2, TLR-4 and TLR-5 on cardiomyocytes by *Staphylococcus aureus* peptidoglycan, *E. coli* lipopolysaccharide, and *Salmonella typhimurium* flagellin, respectively, has been shown to result in decreased cardiomyocyte contractility [8]. However, studies with flagellin demonstrated that TLR engagement did not induce myocardial cell death *in vivo* and that these negative effects on contractility were reversible [9]. Pneumococcal cell wall has been shown to enter cardiomyocytes in a Platelet-activating factor receptor (PAFR) dependent and TLR-2 independent manner and negatively impact contractility of intact mouse and rat hearts without death of cardiomyocytes [10]. Thus, innate immune responses to a range of bacterial components can alter cardiac function transiently but do not appear to induce death of cardiomyocytes or explain the persistence of cardiac dysfunction following acute disease.

As the leading cause of bacterial meningitis [11], the host-pathogen interactions for *S. pneumoniae* occurring at the blood brain barrier have been extensively studied. It is known that bacterial translocation across cerebral vascular endothelial cells is dependent on the binding of the bacterial adhesin Choline binding protein A (CbpA) to endothelial cell Laminin receptor (LR) followed by ligation of phosphorylcholine (ChoP) on the bacterial cell wall to PAFR [12], [13]. These interactions result in the uptake of the bacteria in vesicles and their transport to the basolateral surface of the cell so as to translocate bacteria from the blood into the brain. In the lungs and central nervous system, host cell damage is mediated by pneumolysin, a thiol-activated cholesterol dependent pore-forming toxin that is cytolytic at high concentrations but induces apoptosis at low concentrations [14], [15]. Additional tissue damage may occur as a result of TLR-2 activation by pneumococcal cell wall, which results in profuse cytokine production, immune cell infiltration, and in some instances cell death [10], [16], [17].

Herein, we explored the possibility that *S. pneumoniae* directly damages the heart during invasive pneumococcal disease (IPD) and this contributes towards the occurrence of an adverse cardiac event. We describe the novel observation of non-purulent microscopic lesions (i.e. microlesion) filled with pneumococci within the myocardium and describe the molecular basis for *S. pneumoniae* invasion of cardiac tissue and cardiomyocyte cell death within the lesion. Our findings suggest a previously unrecognized pathological aspect of pneumococcal infection that may help to explain the greater incidence of adverse cardiac events in adults with severe IPD and is potentially vaccine preventable. Decreasing the morbidity and mortality associated with pneumococcal CAP in the aged is particularly critical, as by 2050, 20% of the world population will be >65 years old and as such highly susceptible to CAP and IPD [18].

## Results

### IPD is associated with myocardial damage and alterations in cardiac electrophysiology

**Figure 1:**
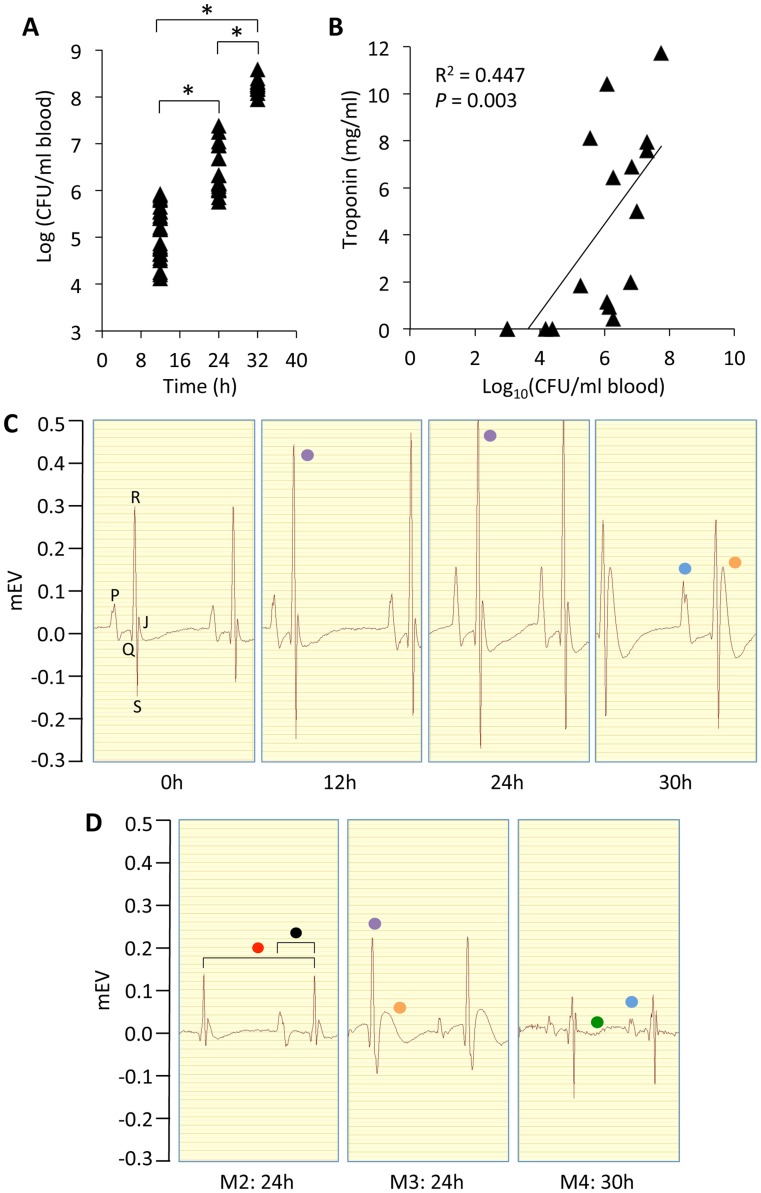
IPD is associated with alterations in cardiac electrophysiology and heart damage. **A)** Bacterial titers in blood of mice at 12 (n = 24), 24 (n = 17), and 30 (n = 11) h following intraperitoneal challenge with 10^3^ CFU of *S. pneumoniae*, strain TIGR4. *P<0.05 by two-tailed Student's *t*-test. **B)** Regression analysis of blood bacterial titers and cardiac troponin-I concentrations at various time points following intraperitoneal challenge with TIGR4 (n = 16). Statistical analysis was done using a Pearson correlation coefficient calculator. **C)** Limb-lead electrocardiogram (ECG) tracings from a single mouse prior to and following intraperitoneal infection at 0, 12, 24 and 30 h. Letters at 0 h identify the corresponding ECG waves. **D)** ECG tracings obtained from 3 representative mice (Mouse [M] 2–4) 24–30 h post infection highlighting the variety of arrhythmias observed among the infected mice (n = 8 for 0, 12, and 24 h; n = 6 for 30 h). The ECGs of control saline treated mice showed no electrical disturbances despite repeated anesthesia and ECG measurement (n = 2; see **Fig. S1**). Note in panels C and D the pronounced bifurcated P-wave (blue dot), the early compensatory increase and then reduced R wave at late time points (purple dot), the presence of a J-wave (orange dot), the elongated intervals for contraction (red dot), PQ wave (black dot) and fibrillation (green dot). ECG tracings were acquired at 200 kHz using the 100B electrocardiogram data acquisition system (iWorx) with mice under isoflurane anesthesia. **Fig. S1** shows an extended ECG rhythm strip for these infected mice.

Challenge of BALB/c mice with *S. pneumoniae* strain TIGR4 via the intraperitoneal route resulted in a linear increase in bacterial burden in blood from 12 h to 30 h post-infection and led to severe IPD (**Fig. 1A**). To test if myocardial tissue damage was incurred during IPD, serum samples collected at various time points were tested for cardiac troponin as a function of the density of *S. pneumoniae* in the blood. A significant positive correlation was observed between bacterial titers and this clinical marker of cardiac injury (**Fig. 1B**). To assess whether alterations in cardiac electrophysiology accompanied cardiac injury, we performed limb-lead ECG analysis prior to and during experimental infection. All infected mice showed initial compensatory alterations, followed by progressive aberrant changes in cardiac electrophysiology (**Fig. 1C** **, Fig. S1**). Uninfected control mice had normal cardiac electrophysiology despite repeated exposure to anesthesia through 48 h (**Fig. S1**). Electrophysiological abnormalities observed during infection included a compensatory increased and then reduced R wave indicating stronger and then weaker contractions, the development of a bifurcated P-wave and prolonged PQ and PR interval indicating disruption of the conduction path from the sino-atrial node and suggestive of multifocal atrial rhythms, and the chaotic conduction of electrical signals indicative of a damaged conduction system (**Fig. 1C–D**). Of note, considerable variability in regards to the specific electrophysiological abnormality observed for each mouse was observed (**Fig. 1D** **, Fig. S1**).

### Cardiac microlesions form as the result of IPD

**Figure 2:**
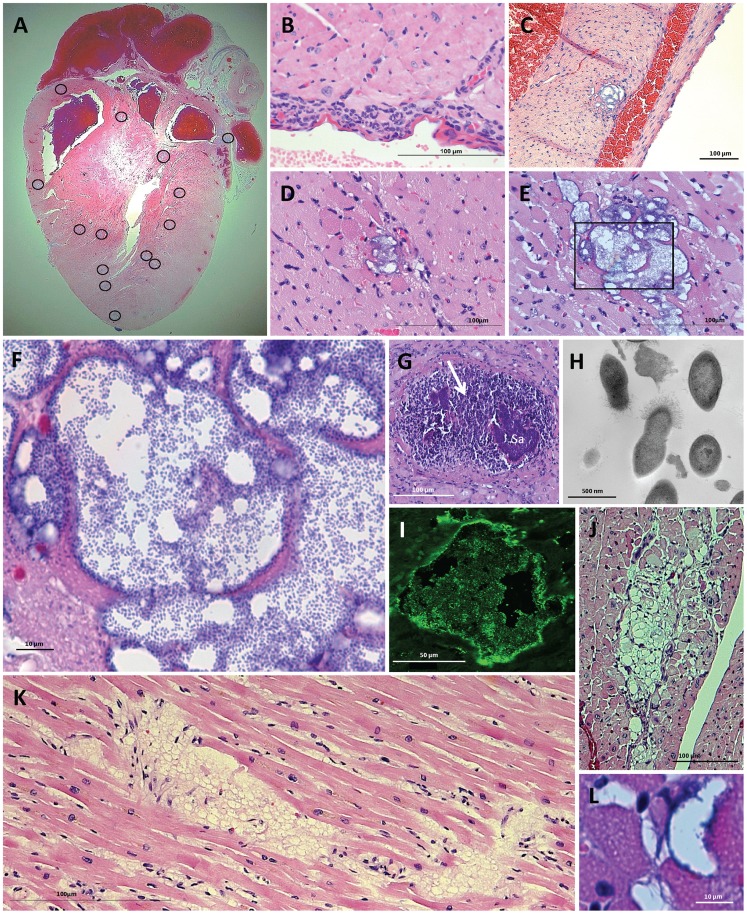
Cardiac lesions form as the result of IPD. H&E stained cross section of a heart obtained from a BALB/c mouse 30 h post-intraperitoneal challenge with TIGR4. **A)** Cardiac microlesions were randomly distributed throughout the mouse myocardium. The circled regions demarcate lesion areas. **B)** Pericarditis was observed in rare mice at 30 h post infection. **C)** Cardiac microlesions were often observed to be adjacent to blood vessels. **D&E)** Representative images of cardiac microlesions seen at 24 h (n = 8) and 30 h (n = 11) post infection, respectively. **F)** Higher powered magnification of the 30 h cardiac microlesion shows *S. pneumoniae* bacterial aggregates within the microlesion. **G)** As a point of contrast, in mice infected *i.p.* with *Staphylococcus aureus* (Sa) abscesses were large and characterized by a robust neutrophil response (white arrow). Tissue sample a gift from Dr. Eric Skaar, Nashville, TN. **H)** Transmission electron microscopy image of cardiac lesion indicates that the bacteria within have diplococcal morphology. **I)** Immunofluorescent detection of the bacterial capsule (serotype 4) confirmed that the granular bodies are *S. pneumoniae*. **J)** Representative cardiac lesion seen in the heart of 3 SIV-infected macaques that had succumbed to experimental pneumococcal challenge despite antimicrobial therapy. Similar lesions were absent in the hearts of macaques that cleared the infection (n = 2). **K)** Cardiac lesion detected in heart of a human adult that had succumbed to IPD. Lesions were observed in 2 of 9 human heart samples. **L)** Cardiac microlesion from a mouse with IPD that had been treated with ampicillin beginning at 30 h post-infection. Cardiac section was collected 12 hours after initiating antimicrobial therapy (n = 4).

**Figure 3:**
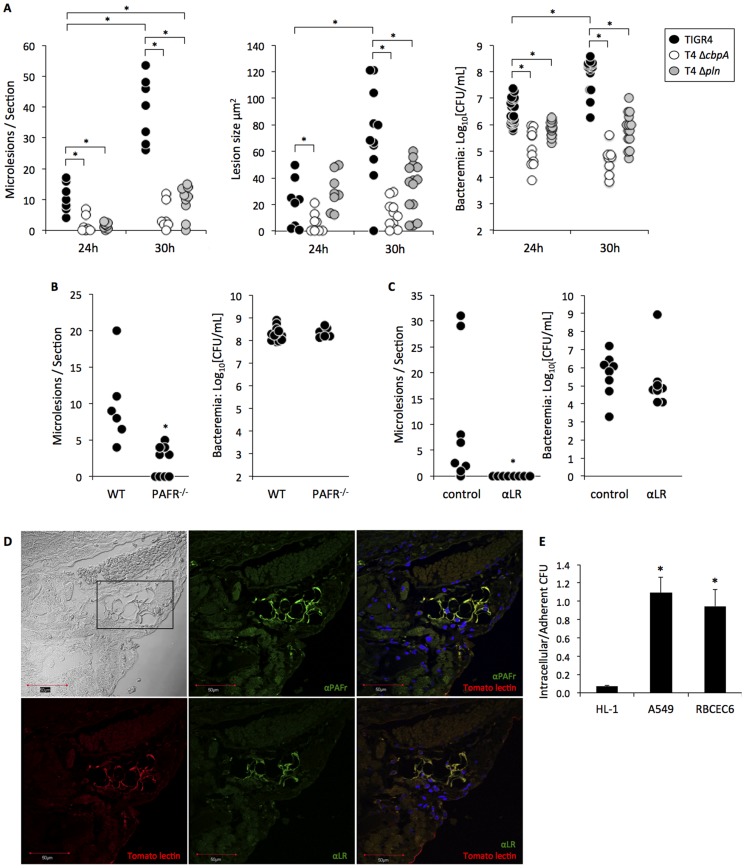
Lesion formation is dependent on the host protein PAFR and the bacterial adhesin CbpA. **A)** Total counts, size of lesions, and bacterial burden in BALB/c mice infected with TIGR4 (24 h n = 8; 30 h n = 12), T4 Δ*cbpA* (24 h n = 8; 30 h n = 13), and T4 Δ*pln* (24 h n = 8; 30 h n = 15) post-infection. **B)** Counts of cardiac lesions and bacterial burden found in sections from TIGR4 infected wild-type C57BL/6 (n = 6, n = 13, respectively) and PAFR^−/−^ (n = 9, n = 6, respectively) mice. **C)** Cardiac lesions and bacterial titers in the blood in TIGR4 infected BALB/c mice following passive immunization with monoclonal antibodies against LR (anti-LR n = 8) or with an isotype control (n = 8). **D)** Immunofluorescence microscopy of a cardiac section treated with FITC conjugated anti-PAFr or anti-LR antibodies in addition to tomato lectin that is selective for vascular endothelial cells. DAPI was used to stain nuclei. Top left image was taken under bright field with the rectangle indicating the location of cardiac blood vessels. Note that on overlaid images of the same tissue section PAFR and LR were found primarily on the vascular endothelial cells and not on cardiomyocytes. **E)** Comparison of pneumococcal invasion rates into rat HL-1 cardiomyocytes, human type II pneumocytes (A549) and rat brain endothelial (RBCEC6) cell lines. The graph represents the ratio of invasive over adherent CFUs (n = 3, each with 4 replicates). Statistical analysis on panels A–C and E was performed using a non-parametric Mann-Whitney rank sum test; *P<0.05.

When the hearts from BALB/c mice with IPD were examined for pathology, we observed the presence of microscopic lesions (microlesions) randomly distributed throughout the ventricular myocardium (**Fig. 2A**). These were distinct from myocarditis and pericarditis that were also occasionally observed (**Fig. 2B**). In many instances, IPD microlesions were adjacent to cardiac blood vessels suggesting cardiac tissue invasion might have arisen by penetration or migration of the bacteria through the vascular endothelium (**Fig. 2C**). Lesions were characterized by the expansion of the interstitium between cardiomyocytes, extracellular vacuolation, the apparent loss of cardiomyocytes, and the stark absence of infiltrating immune cells within the lesion and surrounding tissue (**Fig. 2C–F**). IPD microlesions were highly distinct from the purulent cardiac abscesses that develop when mice are infected with *Staphylococcus aureus* (**Fig. 2G**) [19]; in particular being considerably smaller in size and lacking the prolific infiltration of immune cells. Using high power light microscopy (**Fig. 2F**) and transmission electron microscopy (**Fig. 2H**), bacteria with diplococcal morphology could be seen within microlesions. Of note, although some diplococci were detected within dying cardiomyocytes immediately adjacent to the lesions, the bulk of bacteria were extracellular (**Fig. 2F**). Immunofluorescent imaging using anti-capsular antibody was confirmatory for *S. pneumoniae* (**Fig. 2I**). Microlesions were not detected prior to 24 h following intraperitoneal infection and the number and size of microlesions dramatically increased between 24 h to 30 h (**Fig. 2D–E** **,** **Fig. 3A**) when mice had ∼10^6–7^ and >10^8^ CFU/mL in their blood, respectively.

Cardiac microlesion formation also was observed in C57BL/6 mice infected with TIGR4 (**Fig. 3B**), as well as in BALB/c mice infected with serotype 2 strain D39. For D39, the number of microlesions observed at 30 h (2.34±0.41 lesions/cardiac section; n = 3) was lower than TIGR4 (39.3±9.9 lesions/cardiac section; n = 8, **Fig. 3A**). This may be due to speed that the mice succumbed to D39 infection (only 3 of 9 infected mice survived to 29 h), precluding sufficient time for the microlesions to develop. Importantly, mice infected with TIGR4 via the intratracheal route also developed cardiac microlesions (6.6±3.1 lesions per cardiac section; n = 5). Thus, lesion formation occurred as a result of severe disease and was not restricted by the challenge route. In mice infected with TIGR4, microlesions were not detected in the infected kidneys, livers, or spleens (n = 12). We did however detect a single microlesion in a mouse gastrocnemius muscle at 30 h. Of note, this lesion also lacked the infiltration of immune cells (**Fig. S2**).

To determine if lesions formed in non-human primates, we examined cardiac sections from 3 simian immunodeficiency virus (SIV)-infected rhesus macaques that had succumbed to experimental serotype 19F pneumococcal pneumonia [20]. In these primate experiments, 3 of 23 macaques succumbed to IPD within one week of infection, despite antimicrobial therapy, and all 3 of these animals had cardiac lesions similar in size and with vacuolar morphology. They were distinct from those seen in the mice due to the absence of visible pneumococci (**Fig. 2J**). Two animals that were infected with *S. pneumoniae*, but did not develop fulminate disease, were taken to necropsy one month after bacterial challenge due to evidence of progressive SIV disease. Cardiac lesions similar to those in macaques that died as a result of IPD were not seen in these two animals.

### Evidence of cardiac damage during IPD in humans

We also examined cardiac sections from 9 adults who had succumbed to IPD despite critical care intervention. In heart sections from 2 individuals, vacuolar lesions were observed (**Fig. 2K**). Similar to the experimentally infected macaques that had died of IPD, these lesions also did not contain pneumococci. To determine if the absence of pneumococci in the rhesus macaque and human cardiac lesions was due to the antimicrobial therapy received during critical care, we infected mice with *S. pneumoniae* and intervened 30 h post-infection with high-dose ampicillin therapy. As early as 12 h after administering the antibiotic, we observed cardiac microlesions that were now largely devoid of bacteria yet maintained their vacuolar appearance (**Fig. 2L**).

### Microlesion formation is dependent on host LR and PAFr and the bacterial adhesin CbpA

*S. pneumoniae* translocation across the vascular endothelium requires at least two interactions: the adhesin CbpA binds to host LR and cell wall ChoP binds to host PAFR [12], [21]. Using CbpA deficient pneumococci and PAFR^−/−^ mice, we observed a requirement for these two proteins in cardiac microlesion formation in BALB/c (**Fig. 3A**) and C57BL/6 (**Fig. 3B**) mice, respectively. In addition to serving as an adhesin, CbpA binds to serum Factor H and inhibits complement deposition [22]. Thus, bacterial titers in mice infected with CbpA deficient pneumococci were lower than the WT controls, as expected (**Fig. 3A**). To address the possibility that reduced microlesion formation was due to this lower bacterial load, mice were passively immunized with monoclonal antibody against LR prior to TIGR4 intraperitoneal infection. Antibodies against LR completely blocked cardiac microlesion formation without negatively affecting levels of pneumococci in the blood (**Fig. 3C**). Likewise, no differences in bacterial titers in blood were seen in the PAFR^−/−^ mice infected with TIGR4 versus WT mice (**Fig. 3B**). Thus, disruption of CbpA/LR and ChoP/PAFR interactions *in vivo* inhibited cardiac microlesion formation.

Using immunofluorescent microscopy, we subsequently determined that LR and PAFR were robustly expressed by endothelial cells of vessels throughout the heart but were nearly absent in cardiomyocytes (**Fig. 3D**). This observation was consistent with the low permissiveness of HL-1 cardiomyocytes for pneumococcal invasion *in vitro* in comparison to RBCEC6 rat brain vascular endothelial and A549 human type 2 pneumocyte cell lines (**Fig. 3E**). Thus, high PAFR and LR expression on vascular endothelial cells coupled with low expression on cardiomyocytes is a potential explanation for why the pneumococcus could translocate into the myocardium, yet the bulk of these bacteria were found to be extracellular in cardiac tissue.

### Cardiomyocytes undergo cell death following exposure to pneumolysin

**Figure 4:**
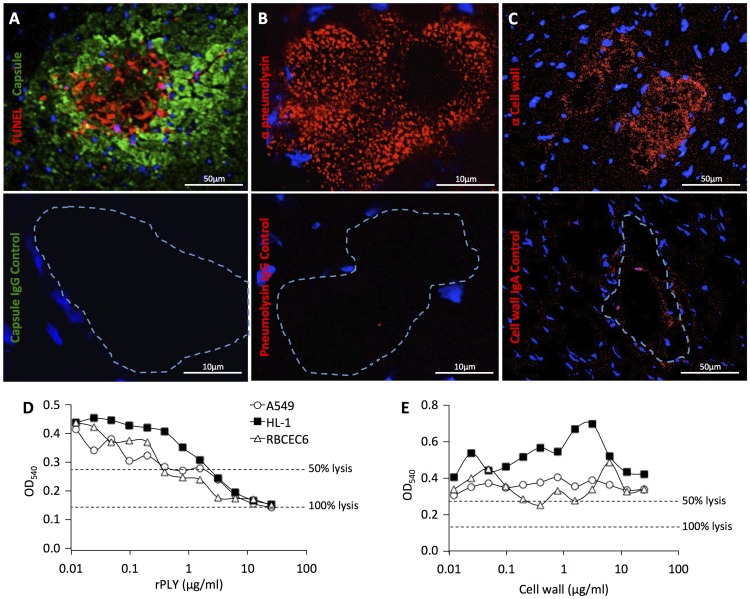
Effect of pneumolysin on cardiomyocyte viability. **A)** Immunofluorescent TUNEL (red) staining of cardiac microlesions from BALB/c mice 30 h following intraperitoneal infection. Pneumococci were detected using antibodies against serotype 4 capsular polysaccharide (green) and cardiomyocyte nuclei stained with DAPI. **B)** Detection of pneumolysin (red) in a microlesion using anti-pneumolysin monoclonal antibody. **C)** Detection of pneumococcal cell wall (red) in microlesions by immunohistochemistry using TEPC-15 an IgA monoclonal antibody against cell wall. For panels **A–C)** fluorescent microscopy using the corresponding control antibody is shown immediately below. Dashed line demarcates the site of the lesion. Vybrant MTT Cell Proliferation Assay was used to determine cell viability of HL-1, A549, and RBCEC6 cells following their exposure to **D)** recombinant pneumolysin (rPLY) or **E)** purified pneumococcal cell wall. Experiments were done 3 times each with 4 replicates. Shown are the results from single, representative experiments.

TUNEL staining indicated the presence of dead or dying cardiomyocytes during early microlesion formation and at the leading edge of mature lesions (**Fig. 4A**). Pneumolysin, the *S. pneumoniae* pore-forming toxin, was localized at the microlesion site using immunofluorescent microscopy (**Fig. 4B**) as was pneumococcal cell wall (**Fig. 4C**), the latter which we have previously shown inhibits cardiac contractility [10]. *In vitro* studies with A549, RBCEC6 and HL-1 cells indicated that pneumococcal attachment and invasion alone did not contribute in a meaningful fashion to host cell death (**Fig. S3**). Yet, *in vitro* HL-1 cardiomyocytes were susceptible to killing with recombinant pneumolysin (**Fig. 4D**), but not with purified cell wall (**Fig. 4E**). Mice infected with a pneumolysin deficient mutant developed significantly fewer and much smaller lesions than the control (**Fig. 3A**). Similar to the CbpA mutant, the pneumolysin mutant did not replicate in the blood to the same levels as wild type. Thus, leaving open the possibility that the absence of microlesions was instead due to the decreased bacterial burden. Retro-orbital injection of mice with a bolus of recombinant pneumolysin (n = 2), purified pneumococcal cell wall (n = 2), or both together (n = 7), failed to cause microlesion formation after 24 h despite considerable signs of damage and inflammation within the cardiac vasculature such as the sloughing of vascular endothelial cells and the presence of adherent leukocytes.

Pneumolysin triggers activation of the NLRP-3 inflammasome [23], which in turn results in the secretion of active IL-1β and in some instances death by pyroptosis [24]. In the lungs and central nervous system, pneumolysin induced IL-1β and cell death have been shown to contribute to the inflamed tissue state and the recruitment of immune cells during pneumococcal infection [23], [25]. Consistent with the absence of immune cell infiltration at cardiac microlesion sites, immunohistochemistry for IL-1β was negative at the lesion sites and IL-1β was not detected in the supernatant of HL-1 cardiomyocytes exposed to pneumolysin (n = 4) or HL-1 cells infected with live bacteria (n = 4) after 2, 4 and 8 h. Likewise, mice deficient in caspase-1 formed lesions similar in morphology at 30 h to those of wildtype mice, albeit >3-fold more frequently in number (WT n = 6, 9.75±2.5 microlesions/section; Caspase-1 KO n = 6, 33.33±8.8 microlesions/section; *P* = 0.026). This may have been due to greater level of bacteremia experienced by the IL-1β deficient mice (WT n = 13, 2.47×10^8^±4.36×10^7^ CFU/mL blood; Caspase-1 KO n = 6, 7.82×10^8^±3.18×10^8^ CFU/mL blood; *P* = 0.012).

### Antibodies against CbpA and pneumolysin protect mice against lesion formation

**Figure 5:**
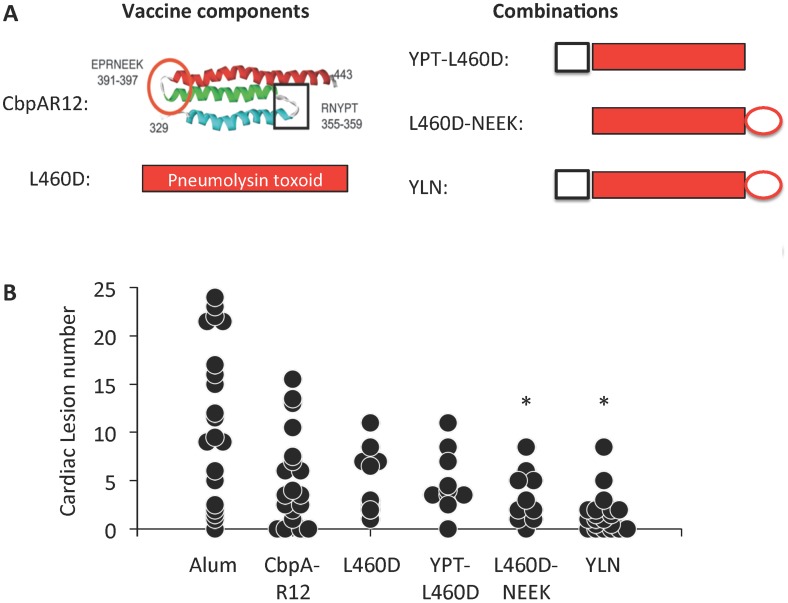
YLN immunized mice are protected against lesion formation. **A)** Top left: Schematic representation of the anti-parallel helices of R domains of CbpA. Square: binding site for polymeric immunoglobulin receptor showing amino acids at the turn (i.e. YPT); Circle: binding site for LR showing amino acids at the turn (i.e. NEEK). Top right: Schematic representation of various fusion protein derivatives of CbpA and the pneumolysin toxoid L460D used for vaccination. YLN is identified as composed of YPT-L460D-NEEK in our studies. **B)** Cardiac lesion number per individual mouse (circle) at 30 h post infection obtained from immunized mice. Experimental cohort size: Alum = 20; CbpA-R12 = 19; L460D = 10; YPT-L460D = 10; L460D-NEEK = 10; YLN = 20. Asterisks denote a statistical significant difference versus the alum control. Statistical analysis was done using Kruskall-Wallis a One-way ANOVA on Ranks.

Given the presumptive critical roles for CbpA and pneumolysin in cardiac microlesion formation, we subsequently tested whether antibodies against these proteins, derived by immunization of mice with individual and fused protein constructs, afforded protection against cardiac damage. More specifically, we tested the pneumolysin toxoid L460D [26], recombinant R1 domain of CbpA that contains the LR and polymeric immunoglobulin receptor binding domains of CbpA (CbpA-R12) [27], and constructs of L460D containing fused peptides from CbpA corresponding to the LR (i.e. NEEK) and the polymeric immunoglobulin receptor (i.e. YPT) binding motifs (**Fig. 5A**) [28]. In humans, the YPT motif of CbpA binds to polymeric immunoglobulin receptor in the nasopharynx [29]. We included the constructs containing the YPT motif as a way to discern if antibodies against CbpA, but not to the LR binding domain, were sufficient to prevent microlesion formation. All mice immunized with these constructs developed high antibody titers to pneumolysin, CbpA, or both, as expected based on their immunogen composition (**Fig. S4A**). In this instance, to avoid early clearance due to pre-existing antibody, a higher bacterial challenge (10^5^ CFU) was used to ensure high and equivalent bacterial titers in the blood during the first 24 h (**Fig. S4B**). Mice immunized with CbpA-R12, L460D, and YPT-L460D did not reach statistical significance versus the alum control. In contrast, mice immunized with the L460D constructs bearing the NEEK domain, L460D-NEEK or YLN, had significantly reduced microlesion formation versus the alum control (**Fig. 5B**).

### Cardiac microlesion sites are characterized by immune cell infiltration and collagen deposition during the convalescent stage

**Figure 6:**
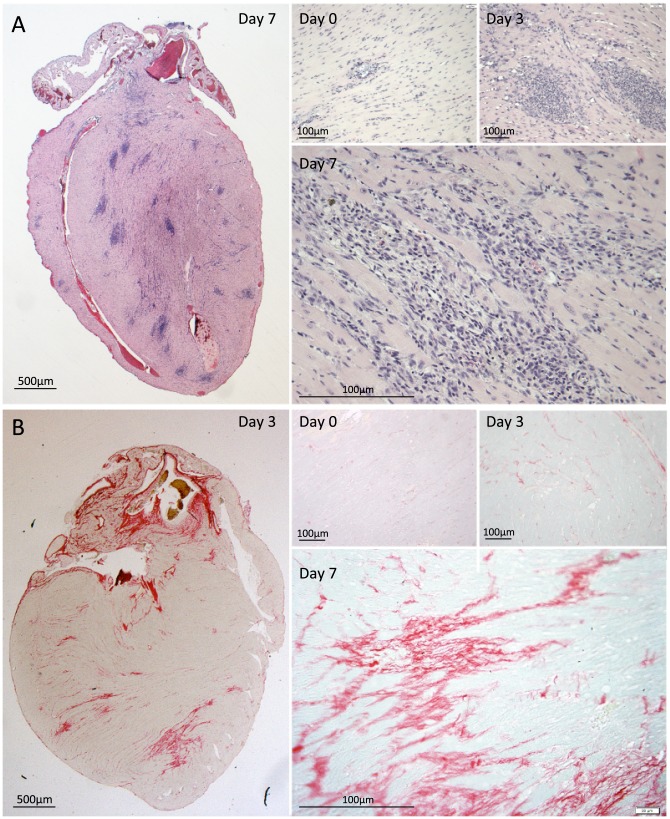
Immune cell infiltration and collagen deposition following antimicrobial therapy. BALB/c mice were infected with *S. pneumoniae* and beginning at 30 h treated with ampicillin for rescue. **A)** Representative H&E stained cross sections of hearts from BALB/c mouse at time when ampicillin treatment was initiated as well as 3 and 7 days post-infection. Note that former microlesion sites are now characterized by robust immune cell infiltration. **B)** Heart sections were also stained with Picrosirius Red to visualize collagen deposition.

We sought to determine how cardiac microlesions resolved following successful antimicrobial therapy. To do this we examined hearts from mice rescued from death with high-dose ampicillin begun at 30 h post-infection. In these mice, blood samples were culture negative 12 h after ampicillin was begun, yet the survival rate was 31.7% (n = 41). In sharp contrast to the lesions before treatment, robust immune cell infiltration at distinct focal sites distributed throughout the myocardium was now observed at day 3, 42 h following the start of antimicrobial therapy, and this persisted through day 7 (**Fig. 6A**). At day 3, the vacuolation characteristic of the microlesions remained discernible in some instances, although visible pneumococci were now completely absent. Based on morphological criteria, immune cells at microlesion sites appeared to be a mixed population of neutrophils and macrophages. Following antibiotic therapy, cardiac inflammation persisted through day 7 with the appearance of collagen in resolving lesions (**Fig. 6B**). These changes were similar to the scarring and remodeling that is known to occur after myocardial infarction [30]–[34].

## Discussion

Despite over a century of investigation of IPD-related complications, this is first report to suggest that pneumococcal invasion of myocardial tissue may occur during IPD. Cardiac microlesion formation can contribute to cardiac dysfunction by physical interruption of conduction pathways, cardiomyocyte death due to pneumolysin, and loss of contractility by the release of cell wall [1]. Cardiac remodeling as a result of collagen deposition is also a viable explanation for the increased mortality rates that are seen in convalescent individuals who have experienced pneumococcal CAP for up to one-year post-infection [4].

*S. pneumoniae* cardiac microlesions were highly distinct from typical Gram-positive abscesses in that they lacked the profuse infiltration of immune cells [19]. They were also distinct from purulent exudate that characterizes pneumococcal infections of the lung and brain. Importantly, when we observed pericarditis (**Fig. 2B**), immune cells were present, suggesting that the absence of an immune cell response may be specific to cardiomyocytes. Yet our observation of a purulent-free lesion within the calf of an infected mouse (**Fig. S2**) instead suggests that this may instead be a phenomena shared by striated muscle cells. The immune response to *S. pneumoniae* is primarily driven by a TLR-2 response to peptidoglycan in cell wall [35]. TLR-2 is found both in skeletal and cardiac muscle, and cardiomyocytes have been shown to respond to *S. aureus* peptidoglycan [8]. Why the host response to cardiomyocyte infection by the pneumococcus is distinct from other tissues or during infection by other pathogens remains unclear. We postulate that the impaired host response to *S. pneumoniae* is, in some fashion, tied to the maintenance of vital cardiac function, but also involves specific host-pathogen interactions that are restricted to the pneumococcus.

Microlesion formation was dependent on CbpA/LR and ChoP/PAFR interactions. These are the same interactions that have been implicated in translocation across the cerebral vascular endothelium during the development of pneumococcal meningitis [12], [13]. Most respiratory tract pathogens, including *Haemophilus influenzae* and *Neisseria meningitidis*, also target LR and PAFR for epithelial and endothelial cell interactions and as such may also be capable of translocation into the myocardium. We have previously shown that statin therapy protects sickle cell mice against fulminate *S. pneumoniae* infection by down-regulating PAFR on endothelial cells and inhibiting the pore-forming activity of pneumolysin [36]. A similar protective effect for statins against cardiac lesion formation during IPD is supported by the fact that individuals on statin therapy who were hospitalized for pneumonia have significantly better post-hospital discharge survival rates than controls [37]; albeit direct evidence that statins impair pneumococcal translocation into the myocardium is lacking. Importantly, the pathophysiology described here is independent of the development of the sepsis syndrome. Microlesions were detected before the onset of sepsis in our experimental model (i.e. 24 h) and this presumably required bacterial translocation into the heart at an even earlier time point. The correlation of lesion formation with duration and intensity of bacteremia, which provides the bacteria with sufficient opportunity to invade the heart, is consistent with what is known regarding the development of meningitis. High-grade persistent bacteremia without translocation of bacteria was insufficient for the development of cardiac microlesions, as evidenced by the absence of lesions in PAFR KO mice and in wildtype mice treated with monoclonal antibodies against LR, both of which had equivalent levels of bacteremia as their respective controls with microlesions. In contrast, high-grade bacteremia when sufficiently prolonged in mice expressing LR and PAFR led to more frequent and larger lesion formation, as evidenced in the Caspase-1 deficient mice. For mice infected with D39, the duration of survival following challenge was most likely insufficient.

Exposure of cardiomyocytes to purified pneumolysin or live *S. pneumoniae* was not associated with release of IL-1β despite the fact that pneumolysin could trigger cell death. The lack of IL-1β indicated cardiomyocyte death was not the result of pyroptosis. Necrotic cell death, such as that caused by membrane lysis, typically elicits a strong inflammatory response due to the release of damage-associated molecular pattern molecules (DAMPs). Along similar lines, necroptosis, a cell-programmed mode of necrosis, has been shown to be involved in ischemia-reperfusion injury of the heart and to be highly inflammatory [38] and Gram-positive pore-forming toxins other than pneumolysin have been implicated as inducers of necroptosis [39], [40]. Yet during acute pneumococcal cardiac microlesion formation, inflammation was decidedly absent. This suggests that instead, pneumolysin triggers immune quiescent apoptosis [41]. Importantly, robust immune cell infiltration was detected at microlesion sites only following antimicrobial therapy. How or why the cardiomyocyte response differs between live versus killed *S. pneumoniae* is unclear. Further studies are required to begin to answer this important question.

Our observation of profuse immune cell infiltration accompanied by collagen deposition after antibiotic therapy, is suggestive that bacterial death after microlesion formation results in cardiac remodeling similar to what is seen following infarction. Such scars have been shown to result in permanent changes in cardiac electrophysiology and function [30]–[34]. Importantly, it is not clear if the class of antimicrobials used to treat IPD would have an impact on cardiac function or the size of the affected region during convalescence. Treatment with cell wall acting antimicrobials, such as ampicillin, results in the lysis of *S. pneumoniae* and this would enhance the release of pneumolysin and cell wall from previously intact pneumococci. In contrast, treatment with antimicrobials that do not result in bacterial lysis, such as macrolides, would presumably limit tissue damage and potentially could improve cardiac outcomes. Along such lines, a reduction in cardiac scarring would also presumably lower the risk for mortality in convalescent individuals. Importantly, immunization of mice with a fusion protein composed of the LR binding domain of CbpA and the pneumolysin toxoid L460D conferred significant protection against microlesion formation. This result supports the critical role for these virulence determinants in microlesion formation and suggests that this form of cardiac damage is potentially vaccine preventable.

Based on our current data, we propose the following model for cardiac microlesion development. During severe invasive disease, pneumococci in the bloodstream engage host LR and PAFR with surface adhesin CbpA and cell wall ChoP residues, respectively. As a result bacteria are translocated into the myocardium. Due to the relative absence of LR and PAFr on cardiomyocytes, the bacteria remain predominantly extracellular, but during replication they release toxic products such as pneumolysin that kill cardiomyocytes and cell wall that inhibits contractility. For as yet unknown reasons, this does not result in the recruitment of immune cells, allowing for further replication of the bacteria and growth of the microlesions. Ultimately, this culminates in altered electrophysiological conductance or contractility that serves as a substrate for acute cardiac events. As such, we propose that during infection microlesion-mediated cardiac damage, increased myocardial demand during infection, ventilation-perfusion mismatch, and the effects of circulating pro-inflammatory factors, together lead towards an adverse outcome in those with IPD. In convalescent animals, profuse immune cell recruitment to the microlesion site occurs accompanied by collagen deposition and possibly permanent scarring. This would most likely exacerbate pre-existing cardiac related problems. Research is merited to determine the true frequency of cardiac microlesions in patients hospitalized with IPD, if modifications in antibiotic therapy improve long-term outcomes, and if prevention of cardiac damage is an indication for vaccination.

## Materials and Methods

### Ethics statement

All mouse experiments were reviewed and approved by the Institutional Animal Care and Use Committees at The University of Texas Health Science Center at San Antonio (protocol #13032-34-01C) and St. Jude Children's Research Hospital (protocol #250). Animal care and experimental protocols adhered to Public Law 89-544 (Animal Welfare Act) and its amendments, Public Health Services guidelines, and the Guide for the Care and Use of Laboratory Animals (U.S. Department of Health & Human Services). Cardiac sections from rhesus macaques were obtained with permission and were remnant from completed and independent investigations performed at Tulane National Primate Research Center [20]. Cardiac sections from individuals who succumbed to IPD were collected during autopsy at Hospital Universitario de Getafe in Madrid Spain from 2000 to 2010, prior to the start of this study. Paraffin-embedded cardiac sections were provided for analysis in a de-identified fashion and work done was determined not to be human subject research by the Institutional Review Board at The University of Texas Health Science Center at San Antonio (protocol #HSC20140389N).

### Mice and macaques

BALB/c, C57BL/6, PAFR^−/−^ [42], Caspase-1^−/−^ (B6N.129S2-*Casp1^tm1Flv^*/J) mice of both sexes were either obtained from The Jackson Laboratory (Bar Harbor, Maine) or from institutional facilities. All mice were used between 10–12 weeks of age. KO mice and their respective WT controls were obtained from the same facility and were raised under similar conditions. Non-human primate studies were conducted on male rhesus macaques (*Macaca mulatta*) of Indian origin at 4 to 6 years of age. All monkeys were infected with SIV Mac251 4 months prior to *S. pneumoniae* challenge [20].

### Bacterial strains and media

Wild type strains used in this study included *S. pneumoniae* serotype 4 strain TIGR4 [43], serotype 2 strain D39 [44], and serotype 19F strain 6319 (ATCC 6319). Isogenic TIGR4 mutants lacking CbpA (Δ*cbpA* ^−^), and pneumolysin (Δ*pln* ^−^) have been previously described [45]. To generate purified pneumococcal cell wall, we used the unencapsulated strain R6 and followed published protocols [10]. *S. pneumoniae* was grown in Todd-Hewitt broth or on blood agar plates at 37°C in 5% CO_2_. Recombinant pneumolysin was purified from transformed *Escherichia coli* and hemolytic activity measured [46].

### Infection and histology

For mouse experiments, exponential phase cultures of *S. pneumoniae* were centrifuged, washed with sterile phosphate-buffered saline (PBS), and suspended in PBS at a final concentration of colony-forming units 1×10^4^ CFU/mL. Mice were anesthetized with 2.5% vaporized isoflurane and injected intraperitoneally (*i.p.*) with 100 µl of the *S. pneumoniae* suspension. Bacterial titers were determined by extrapolation of colony counts from plated serial dilution of tail bleeds. Once sacrificed, the heart and/or other organs were harvested, washed in PBS to remove excess blood, placed into specimen collection cassettes and set into 10% formalin solution and subsequently paraffin embedded. A detailed description of the experimental protocol used for infection of the rhesus macaques is available [20]. Macaques were administered 2×10^6^ CFU of *S. pneumoniae* strain 6319 in 2 mL saline into a subsegment of the right lower lobe using a pediatric fiber optic bronchoscope. These studies were designed to determine the effect of chronic alcohol on lung viral titers and the host response to pneumococcal lung infection. Heart tissue came from animals that expired within the first 4 days due to fulminant bacterial infection or were euthanized after 28 days. Paraffin embedded cardiac samples were sectioned then stained with hematoxylin and eosin (H&E) and/or Gram stained by the University of Texas Health Science Center at San Antonio Histology and Immunohistochemistry Laboratory. Picrosirius Red staining was performed for detection of collagen deposition. Tissue sections were mounted with Permount (Fisher Scientific) mounting solution.

### Antibiotic rescue of septic mice

Mice were infected with 10^3^ CFU of TIGR4. Beginning at 30 hours post-infection, mice were administered ampicillin (20 mg/kg body weight) in saline *i.p.* every 12 hours 3X. Hearts were collected at designated time points and processed for histological examination. Blood was collected from the tail vein, plated, and the plates incubated to confirm bacterial clearance.

### Electrocardiogram analysis and cardiac troponin assay

Limb-lead ECGs were acquired at 200 kHz using the 100B electrocardiogram data acquisition system (iWorx) with mice under 1–2.5% vaporized isoflurane anesthesia in an oxygen mix on a heated surgical platform (Indus Instruments). At designated times, infected mice were euthanized and exsanguinated by cardiac puncture. An aliquot of blood was diluted in saline containing heparin and used to extrapolate bacterial titers from colony counts. The remainder of blood was processed for serum collection. Cardiac troponin in these samples was determined using the mouse Cardiac Tn-I ELISA kit (Life Diagnostics).

### Fluorescent microscopy of tissue sections

Immunofluorescent microscopy was done using both fixed and frozen cardiac sections. Fixed cardiac sections were deparaffinized and rehydrated by placing section in xylene, and subsequent graded ethanol washes. Samples were permeabilized with 10 mM sodium citrate pH = 6 for 10 min, washed with PBS, and blocked with 10% fetal bovine serum (FBS) in PBS for 1 h. Frozen sections on glass slides were fixed in 4% paraformaldehyde, permeabilized in 0.2% Triton X, blocked with 10% fetal bovine serum (FBS) in PBS for 1 h. Cardiac sections were subsequently incubated with either rabbit anti-serotype 4 pneumococcus antiserum (1∶1,000) (Statens Serum Institut), TEPC15 IgA Kappa from murine myeloma (1∶500) (Sigma), rabbit anti-pneumolysin polyclonal antibody (1∶50) (Abcam), anti-laminin receptor monoclonal antibody (1∶200) (Abcam) or anti-PAFR mouse monoclonal antiobdy (1∶500) (Cayman Chemical) antibody, or the respective isotype control antibody at the corresponding dilution. After washing with PBS, sections were covered with 10% goat or BSA containing either goat anti-rabbit FITC conjugated antibody (1∶2,000) (Invitrogen) or donkey anti-rabbit rhodamine conjugated antibody (1∶200) (Millipore). Using the Invitrogen SuperPicTure Kit, pneumolysin could be visualized. Sections were counterstained with Harris hematoxylin solution (Sigma) and mounted using Histomount solution (Invitrogen). To visualize vascular endothelial cells, labeled tomato lectin from *Lycopersicon Esculentum* (Vector Laboratories) was used (1∶1000). DAPI (4′,6-Diamidino-2-Phenylindole, Dilactate) at 5 mg/mL was used for visualization of eukaryotic nuclei. Tissue sections were washed and mounted with FluorSave (Merck Biosciences). For TUNEL analysis of cardiac microlesions, the Millipore ApoptagKit Red In Situ Apoptosis Detection Kit (EMD Millipore Corp.) was used to detect fragmented DNA. Images were acquired using an Olympus FV-1000 confocal system, running the Fluoview 3.1 software (Olympus Corporation) at the University of Texas Health Science Center Optical Imaging Core Facility.

### Transmission electron microscopy

Mouse hearts were excised and placed in phosphate buffered 4% formaldehyde with 1% glutaraldehyde prior to processing. The hearts were prepared for TEM imaging as previously described [47]. Images were generated in the UTHSCSA Electron Microscopy Laboratory using the JEOL 1230 microscope and AMT digital imaging system.

### Cell based assays

HL-1 atrial myocytes (a gift from Dr. W. Claycomb, Louisiana State University, New Orleans, LA) were maintained in Claycomb's medium (JRH Biosciences) supplemented with 10% FBS (JRH Biosciences), 2 mM L-glutamine (Invitrogen Life Technologies), and 0.1 mM norepinephrine (Sigma-Aldrich). Adhesion and invasion assays of A549 lung epithelial cells (ATCC) and RBCEC6 brain endothelial cells with unencapsulated TIGR4 (T4R) were performed as previously described [42]. Cytotoxicity after infection with T4R was determined by measuring LDH (Thermo Scientific) in the cell culture supernatents following a 30 minute incubation for T4R adhesion or a 2 hour incubation for T4R invasion. One non-infected well per assay was used to determine eukaryotic cells/well. The graph represents the average of CFUs that adhered to or invaded each cell. For cell viability experiments, A549, HL-1 and RBCEC6 cells were seeded in a 96 well plate at 3.5×10^5^ cells/mL (200 uL/well) in serum free F12K media (phenol red free). Cells were grown for 24 h at 37°C 5% CO_2_. Recombinant pneumolysin was serial diluted (buffer PBS, 0.1% BSA, 0.15% DTT) 1∶2 with a starting concentration of 25 µg/mL. 50 µL diluted rPLY was added to the cells and the plates were incubated 24 h at 37°C 5% CO_2_. Vybrant MTT Cell Proliferation Assay was used to determine cell viability according to manufacturer's protocol (Molecular Probes). Absorbance was read at 540 nm and plotted. Data represent the average of 3 independent experiments with 4 wells per pneumolysin dilution. For experiments testing cardiomyocytes for necroptosis, HL-1 cardiac cells were infected with the T4R strain of *S. pneumoniae* at MOI 0.1, 1, and 10 for 6 hrs+/−30 uM Necrostatin-1 (Alfa Aesar). Cells were stained with Annexin V APC (BD Biosciences) and Propidium Iodide (BD Biosciences) for flow cytometry (BD FACSCanto II) and analysis by FlowJo software (TreeStar Inc.).

### Mouse immunization and challenge

BALB/c Mice were primed (day 1) and boosted (days 14 and 28) *i.p.* with 10 µg protein and 130 µg alum. Serum was obtained (day 35) and IgG titers against pneumolysin and CbpA were determined by ELISA. Mice were challenged (day 50) *i.p.* with 1×10^3^ CFU TIGR4. At 24 h post challenge blood was drawn and plated on blood agar plates to determine degree of bacteremia. Mice were sacrificed at 30 h post challenge and the hearts were harvested for histopathology. For some experiments, mice were injected retro-orbitally with either immunoglobulin-isotype control or 40 µg of monoclonal antibody against 67-kDa laminin receptor (Abcam) [48].

### Statistical analysis

Regression analysis of bacterial titers and troponin levels was performed using a Pearson correlation coefficient calculator. Pair-wise comparisons were performed either using a Student's *t*-test or non-parametric Mann-Whitney rank sum test. For comparisons between 3 or more cohorts a Kruskal-Wallis One Way ANOVA on Ranks was used.

## Supporting Information

Figure S1 ECG tracings from individual mice (M) following intraperitoneal challenge with *S. pneumoniae*. Note the onset of aberrant electrophysiology. Saline challenged controls (C) showed no disturbances through 48 h despite identical anesthesia treatment and repeated ECG measurements.
(PDF)

Figure S2 Microlesion lacking immune cell infiltrates and filled with *Streptococcus pneumoniae* found in the gastrocnemius muscle of a mouse 30 h after intraperitoneal challenge.
(PDF)

Figure S3 Cell cytotoxicity following pneumococcal adhesion and invasion. Cytotoxicity as measured by release of lactose dehydrogenase in cell culture supernatants following a 30 min incubation for pneumococcal adhesion or a 2 h incubation for invasion. Lysis buffer served as the positive control for 100% cell toxicity while serum-free media was used as a negative control. Assay was performed using 4 wells per sample. Results shown are representative of duplicate experiments.
(PDF)

Figure S4 **A)** Antibody response to pneumococcal protein constructions. Antibody titers to pneumolysin and CbpA in serum of mice as determined by ELISA following their immunization with the designated recombinant constructs. Circles represent values for individual mice. **B)** Levels of bacteremia following pneumococcal challenge. Bacterial titers in the blood of these same mice 24 h after high-dose intraperitoneal injection with 10^5^ CFU TIGR4. Experimental cohort size: Alum = 20; CbpA-R12 = 20; L460D = 10; YPT-L460D = 10; L460D-NEEK = 10; YLN = 20.
(PDF)
