# Supplementary material for: Streptococcus pneumoniae Translocates into the Myocardium and Forms Unique Microlesions That Disrupt Cardiac Function
Source: PLoS Pathog. 2014 Sep 18;10(9):e1004383. doi: 10.1371/journal.ppat.1004383 (PMC4169480; doi:10.1371/journal.ppat.1004383)
Supplement: Figure S1 — ECG tracings from individual mice (M) following intraperitoneal challenge with S. pneumoniae. Note the onset of aberrant electrophysiology. Saline challenged controls (C) showed no disturbances through 48 h despite identical anesthesia treatment and repeated ECG measurements. (PDF) [file ppat.1004383.s001.pdf]

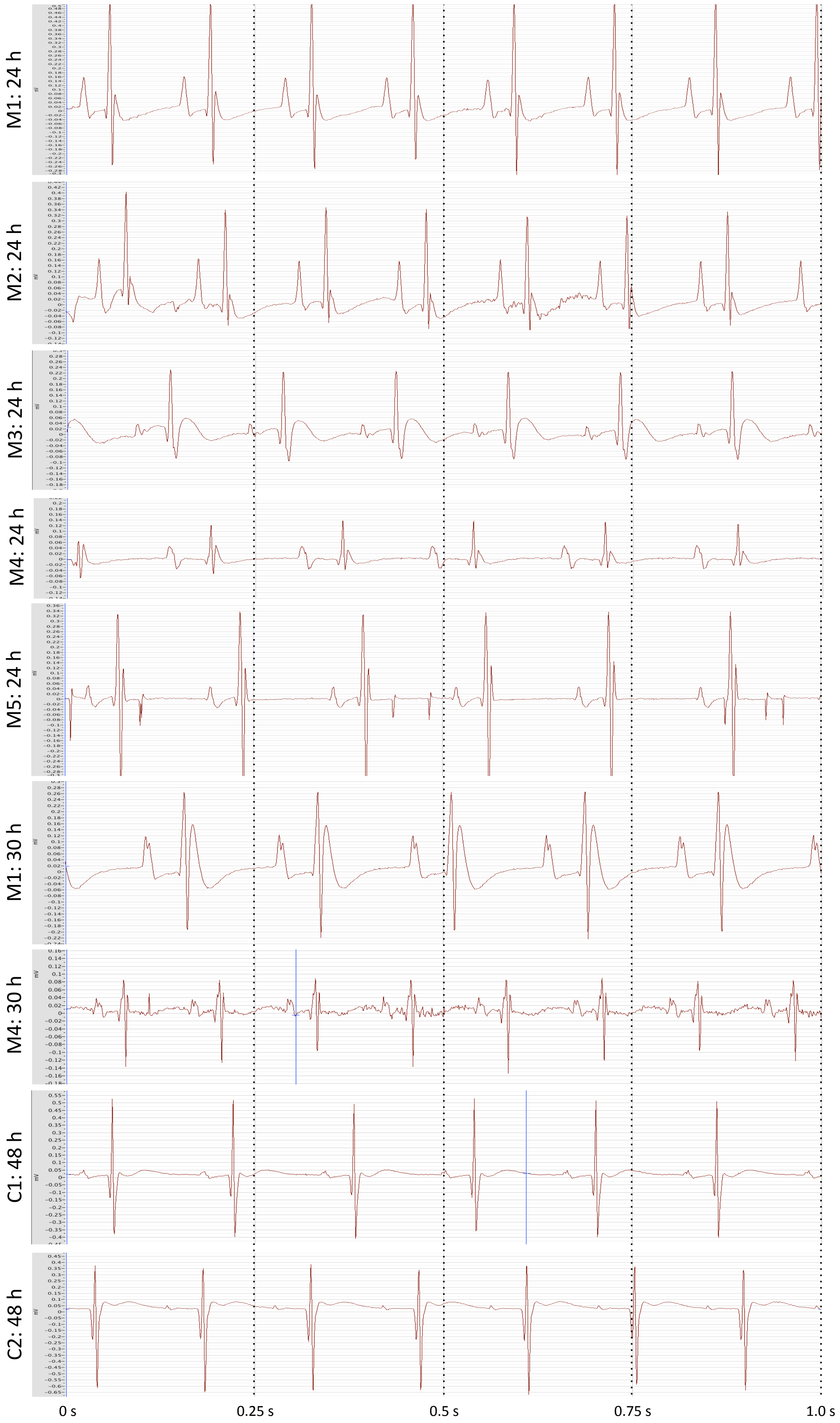

Figure S1. ECG tracings from individual mice (M) following intraperitoneal challenge with *S. pneumoniae*. Note the onset of aberrant electrophysiology. Saline challenged controls (C) showed no disturbances through 48 h despite identical anesthesia treatment and repeated ECG measurements.
