# Supplementary material for: Streptococcus pneumoniae Translocates into the Myocardium and Forms Unique Microlesions That Disrupt Cardiac Function
Source: PLoS Pathog. 2014 Sep 18;10(9):e1004383. doi: 10.1371/journal.ppat.1004383 (PMC4169480; doi:10.1371/journal.ppat.1004383)
Supplement: Figure S2 — Microlesion lacking immune cell infiltrates and filled with Streptococcus pneumoniae found in the gastrocnemius muscle of a mouse 30 h after intraperitoneal challenge. (PDF) [file ppat.1004383.s002.pdf]

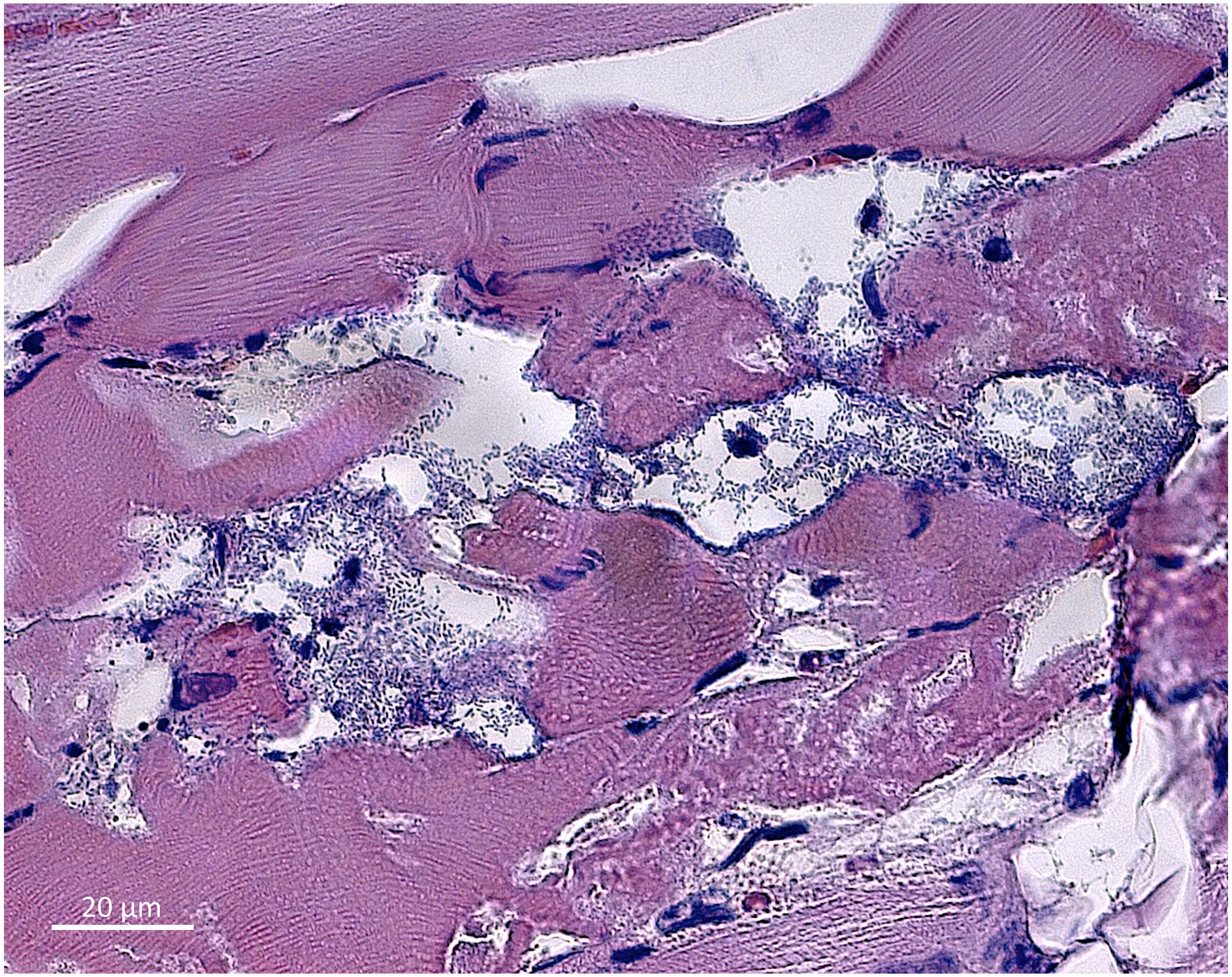

Figure S2. Microlesion lacking immune cell infiltrates and filled with *Streptococcus pneumoniae* were found in the gastrocnemius muscle of a mouse 30 h after intraperitoneal challenge.
