# Supplementary material for: Streptococcus pneumoniae Translocates into the Myocardium and Forms Unique Microlesions That Disrupt Cardiac Function
Source: PLoS Pathog. 2014 Sep 18;10(9):e1004383. doi: 10.1371/journal.ppat.1004383 (PMC4169480; doi:10.1371/journal.ppat.1004383)
Supplement: Figure S3 — Cell cytotoxicity following pneumococcal adhesion and invasion. Cytotoxicity as measured by release of lactose dehydrogenase in cell culture supernatants following a 30 min incubation for pneumococcal adhesion or a 2 h incubation for invasion. Lysis buffer served as the positive control for 100% cell toxicity while serum-free media was used as a negative control. Assay was performed using 4 wells per sample. Results shown are representative of duplicate experiments. (PDF) [file ppat.1004383.s003.pdf]

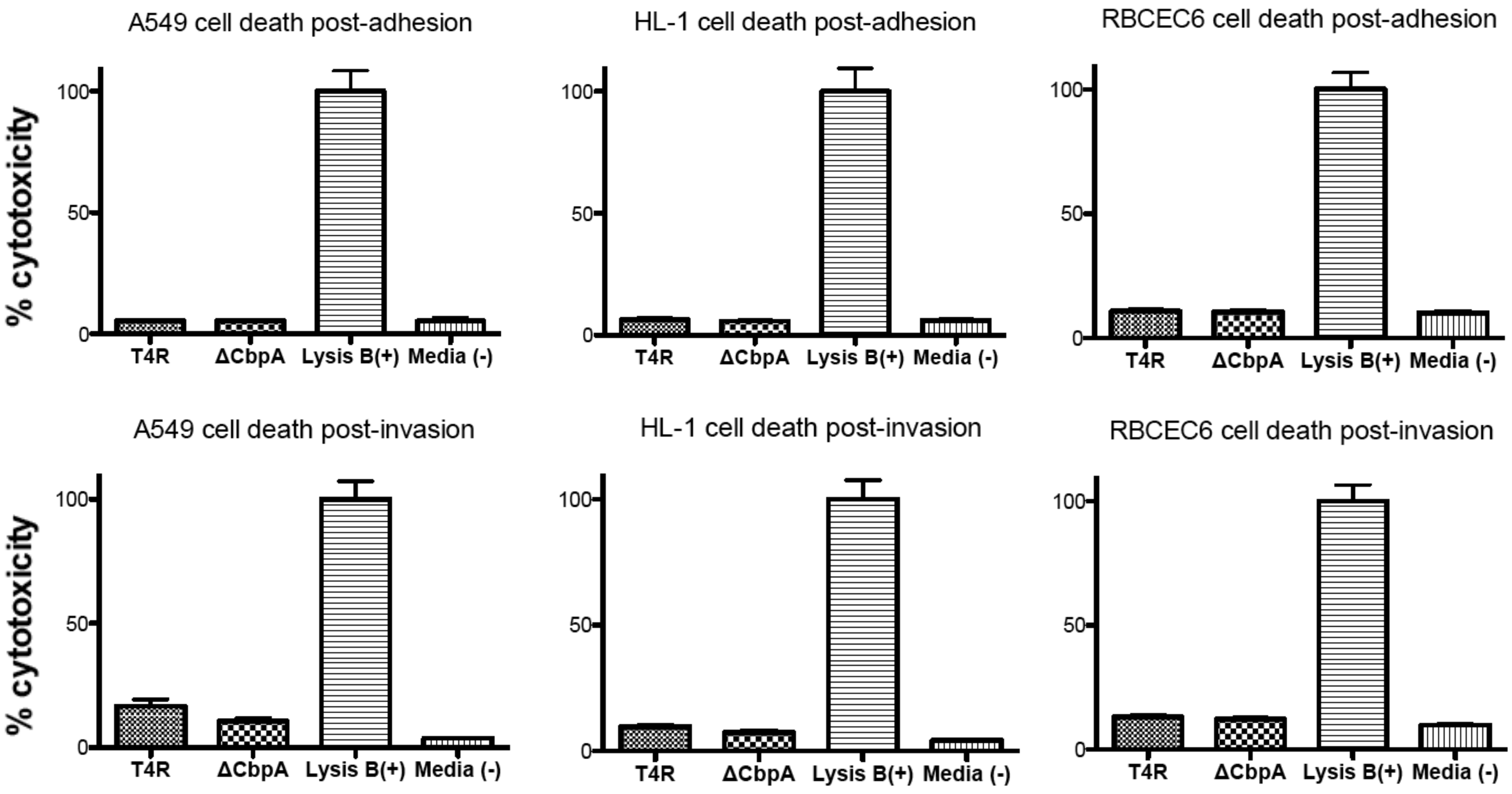

Figure S3. Cell cytotoxicity following pneumococcal adhesion and invasion. Cytotoxicity as measured by release of lactose dehydrogenase in cell culture supernatants following a 30 min incubation for pneumococcal adhesion or a 2 h incubation for invasion. Lysis buffer served as the positive control for 100% cell toxicity while serum-free media was used as a negative control. Assay was performed using 4 wells per sample. Results shown are representative of duplicate experiments.
