# Supplementary material for: Streptococcus pneumoniae Translocates into the Myocardium and Forms Unique Microlesions That Disrupt Cardiac Function
Source: PLoS Pathog. 2014 Sep 18;10(9):e1004383. doi: 10.1371/journal.ppat.1004383 (PMC4169480; doi:10.1371/journal.ppat.1004383)
Supplement: Figure S4 — A) Antibody response to pneumococcal protein constructions. Antibody titers to pneumolysin and CbpA in serum of mice as determined by ELISA following their immunization with the designated recombinant constructs. Circles represent values for individual mice. B) Levels of bacteremia following pneumococcal challenge. Bacterial titers in the blood of these same mice 24 h after high-dose intraperitoneal injection with 105 CFU TIGR4. Experimental cohort size: Alum = 20; CbpA-R12 = 20; L460D = 10; YPT-L460D = 10; L460D-NEEK = 10; YLN = 20. (PDF) [file ppat.1004383.s004.pdf]

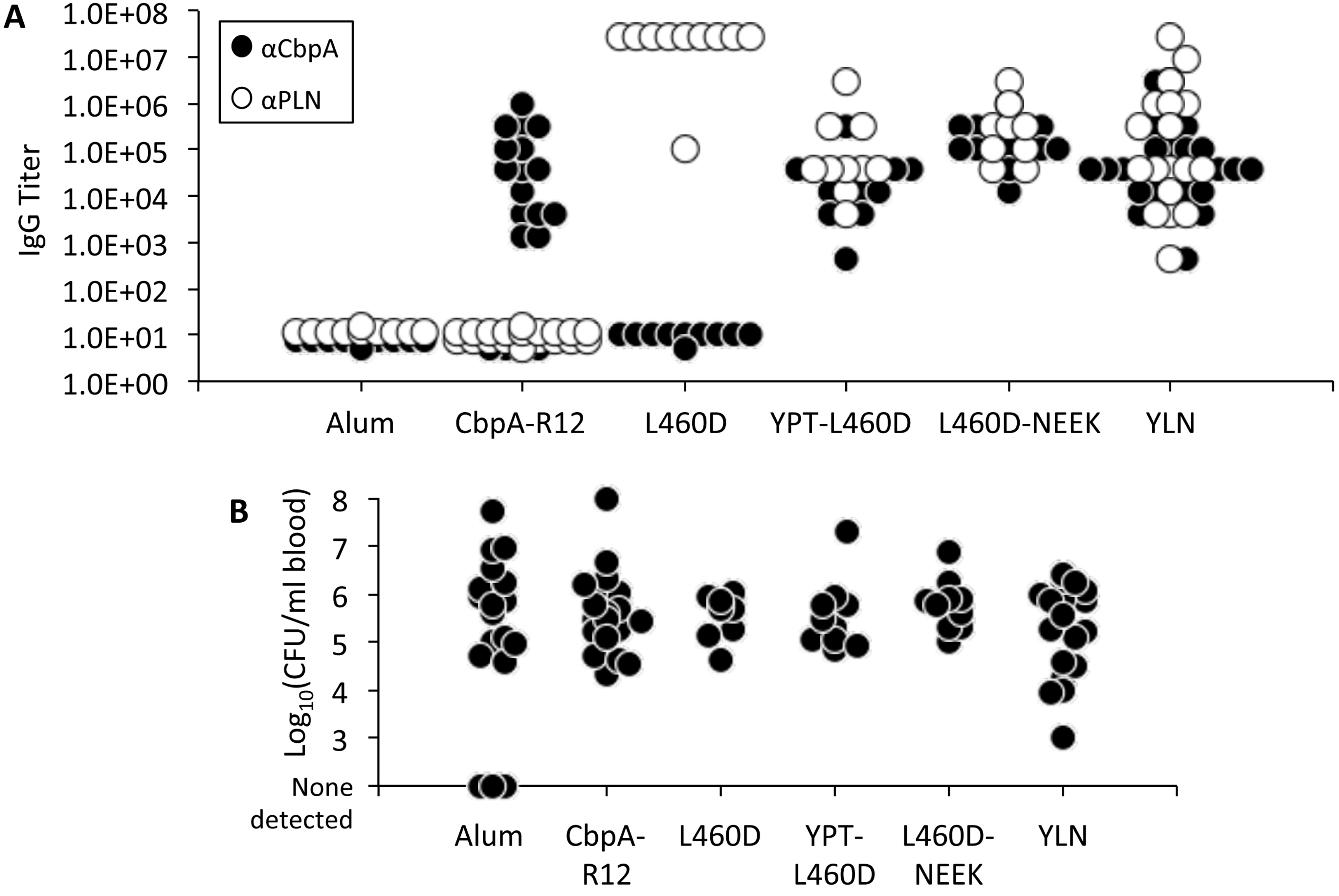

Figure S4. A) Antibody response to pneumococcal protein constructions. Antibody titers to pneumolysin and CbpA in serum of mice as determined by ELISA following their immunization with the designated recombinant constructs. Circles represent values for individual mice. **B) Levels of bacteremia following pneumococcal challenge.** Bacterial titers in the blood of these same mice 24 h after high-dose intraperitoneal injection with 10^5 CFU TIGR4. Experimental cohort size: Alum = 20; CbpA-R12 = 20; L460D = 10; YPT-L460D = 10; L460D-NEEK = 10; YLN = 20.
